# Supplementary material for: SEMA6A drives GnRH neuron-dependent puberty onset by tuning median eminence vascular permeability
Source: Nat Commun. 2023 Dec 7;14:8097. doi: 10.1038/s41467-023-43820-z (PMC10703890; doi:10.1038/s41467-023-43820-z)
Supplement: Supplementary file 3 — Reporting Summary [file 41467_2023_43820_MOESM3_ESM.pdf]

Corresponding author(s): Anna Cariboni, Jeroen Pasterkamp, Alessandro Fantin

Last updated by author(s): Nov 20, 2023

Reporting Summary

Nature Portfolio wishes to improve the reproducibility of the work that we publish. This form provides structure for consistency and transparency in reporting. For further information on Nature Portfolio policies, see our Editorial Policies and the Editorial Policy Checklist.

Statistics

For all statistical analyses, confirm that the following items are present in the figure legend, table legend, main text, or Methods section.

n/a	Confirmed	
☐	☒	The exact sample size (*n*) for each experimental group/condition, given as a discrete number and unit of measurement
☐	☒	A statement on whether measurements were taken from distinct samples or whether the same sample was measured repeatedly
☐	☒	The statistical test(s) used AND whether they are one- or two-sided<br>*Only common tests should be described solely by name; describe more complex techniques in the Methods section.*
☒	☐	A description of all covariates tested
☒	☐	A description of any assumptions or corrections, such as tests of normality and adjustment for multiple comparisons
☐	☒	A full description of the statistical parameters including central tendency (e.g. means) or other basic estimates (e.g. regression coefficient) AND variation (e.g. standard deviation) or associated estimates of uncertainty (e.g. confidence intervals)
☐	☒	For null hypothesis testing, the test statistic (e.g. *F*, *t*, *r*) with confidence intervals, effect sizes, degrees of freedom and *P* value noted<br>*Give P values as exact values whenever suitable.*
☒	☐	For Bayesian analysis, information on the choice of priors and Markov chain Monte Carlo settings
☒	☐	For hierarchical and complex designs, identification of the appropriate level for tests and full reporting of outcomes
☒	☐	Estimates of effect sizes (e.g. Cohen's *d*, Pearson's *r*), indicating how they were calculated

Our web collection on statistics for biologists contains articles on many of the points above.

Software and code

Policy information about availability of computer code

Data collection

For brightfield and confocal image acquisition IS Capture v3.6.7 (TiEsseLab), NIS Elements AR v5.21.03 (Nikon) and ZEN 3.0 Suite (Zeiss) software were used. Western blot images were acquired by using Image Lab v5.2.1 (Biorad) software.

For whole and targeted exome sequencing Nimblegen V2 or Agilent V5 platform and Ilumina HiSeq 2000 Sequencing were used.

Data analysis

Image J v1.52a (NIH) was used for Western blot, median eminence GnRH innervation, median eminence Evans Blue diffusion and testis CYP17A1+ area quantifications.

Prism v8.2.1 (GraphPad) was used for all statistical analyses.

Photoshop CS6 (Adobe) and Fiji v2.3.0/1.53f (NIH) were used for figure preparation.

RStudio v.1.3.1056 was used to analyze scRNA-seq data.

Whole and targeted exome sequencing: BWA-MEM [bwa-0.7.12], Picard tools 1.119, GATK-3.4-46. Open sources: Picard Tools v1.141 (CollectWgsMetrics), gnomAD database v2.0.2. All tools and scripts for processing the WES data can be provided by authors upon request.

Variant pathogenicity was assessed usign the following software: PolyPhen-2 (http://genetics.bwh.harvard.edu/pph2/), SIFT (https://sift.bii.a-star.edu.sg/), CADD (https://cadd.gs.washington.edu/) and REVEL (https://sites.google.com/site/revelgenomics/).

The CryoEM structure of the SEMA6A homodimer in complex with C. sordellii lethal toxin TcsL (PDB ID: 6WTS) was retrieved from the RCSB

Protein Data Bank. Structure preparation procedure was performed using Schrödinger 2020-4 BioLuminate Protein Preparation Tool with the OPLS3e force field. The evaluation of the impact of the mutation on the protein stability was perfumed using Schrödinger 2020-4 BioLuminate Residue Scanning Tool.

For manuscripts utilizing custom algorithms or software that are central to the research but not yet described in published literature, software must be made available to editors and reviewers. We strongly encourage code deposition in a community repository (e.g. GitHub). See the Nature Portfolio guidelines for submitting code & software for further information.

Data

Policy information about availability of data

All manuscripts must include a data availability statement. This statement should provide the following information, where applicable:

- Accession codes, unique identifiers, or web links for publicly available datasets
- A description of any restrictions on data availability
- For clinical datasets or third party data, please ensure that the statement adheres to our policy

All data supporting the findings described in this manuscript are available in the article and its Supplementary Information files. Source data are provided with this paper. All unique materials are readily available from the authors upon request.
The exome sequencing data are available under restricted access due to ethics restrictions on storing and sharing our pediatric patient exome data. These data will have controlled access and will be limited to individuals who enter a research agreement; use of these genomic data will be restricted to those named on the agreement, and exome data will be patient de-identified. Data will be shared after contacting the corresponding author (A.C.: anna.cariboni@unimi.it), who ensures a response and the access to the data within one month.
The SEMA6A variant has been deposited in the ClinVar database under accession code VCV002571587.2 (https://www.ncbi.nlm.nih.gov/clinvar/variation/2571587/).
The single-cell transcriptomic data Tabula Muris (https://tabula-muris.ds.czbiohub.org/) (ref#46) and EC atlas (https://endotheliomics.shinyapps.io/ec_atlas/) (ref#53) used in this study are available in the GEO and EBI ArrayExpress database under accession code GSE109774 https://www.ncbi.nlm.nih.gov/geo/query/acc.cgi?acc=GSE109774) E-MTAB-8077 (https://www.ebi.ac.uk/biostudies/arrayexpress/studies/E-MTAB-8077?query=E-MTAB-8077).
The bulk RNA-seq expression data used in this study were analyzed through BulkECexplorer (https://ruhrberglab.shinyapps.io/BulkECexplorer/) (ref#54), an online resource that surveys 240 publicly available bulk RNA-seq datasets from five human and mouse EC subtypes, including HUVECs and mBECs.
In addition, the following databases were used: gnomAD (https://gnomad.broadinstitute.org/), VarSome (https://varsome.com) and PDB (https://www.rcsb.org).

Research involving human participants, their data, or biological material

Policy information about studies with human participants or human data. See also policy information about sex, gender (identity/presentation), and sexual orientation and race, ethnicity and racism.

Reporting on sex and gender	Sex (collected by participant self-report) for the 100 probands that provided samples for whole exome sequencing was as follows: male = 87, female = 13).
Reporting on race, ethnicity, or other socially relevant groupings	*Please specify the socially constructed or socially relevant categorization variable(s) used in your manuscript and explain why they were used. Please note that such variables should not be used as proxies for other socially constructed/relevant variables (for example, race or ethnicity should not be used as a proxy for socioeconomic status).*<br>*Provide clear definitions of the relevant terms used, how they were provided (by the participants/respondents, the researchers, or third parties), and the method(s) used to classify people into the different categories (e.g. self-report, census or administrative data, social media data, etc.)*<br>*Please provide details about how you controlled for confounding variables in your analyses.*
Population characteristics	All patients were referred to specialist paediatric care centres in central and southern Finland (1982 to 2004). All patients met the diagnostic criteria for self-limited delayed puberty, defined as the onset of Tanner stage G2 (testicular volume >3 ml) at >13.5 year in boys or Tanner stage B2 at >13.0 year in girls (i.e. two SD later than average pubertal development). Chronic illness and undernutrition was excluded by medical history, clinical examination, and routine laboratory investigations. GnRH deficiency was excluded by spontaneous pubertal development at follow-up.
Recruitment	Patients referred with delayed puberty to specialist paediatric care centres in central and southern Finland (1982 to 2004) were identified and offered recruitment. Families of the DP patients were invited to participate, with information about medical history and pubertal timing information obtained from archived growth records and structured interviews.
Ethics oversight	The human fetal tissue was provided by the Joint MRC/Wellcome Trust (grant #099175/Z/12/Z) Human Developmental Biology Resource (www.hdbr.org), with appropriate maternal written informed consent and approval from the Ethics Committee NRES Committee London – Fulham. HDBR is regulated by the UK Human Tissue Authority (HTA; www.hta.gov.uk) and operates in accordance with the relevant HTA Codes of Practice. Fetal tissues from both sources were obtained from terminations at 7 post conception weeks (Carnegie Stage 19). The human study protocol was approved by the Ethics Committee for Pediatrics, Adolescent Medicine and Psychiatry, Hospital District of Helsinki and Uusimaa (570/E7/2003). Ethical approval for human studies was granted by the UK London-Chelsea NRES committee (13/LO/0257). All participants provided written informed consent prior to study participation. The study was conducted in accordance with the guidelines of The Declaration of Helsinki. Participants did not receive compensation.

Note that full information on the approval of the study protocol must also be provided in the manuscript.

Field-specific reporting

Please select the one below that is the best fit for your research. If you are not sure, read the appropriate sections before making your selection.

[X] Life sciences   [ ] Behavioural & social sciences   [ ] Ecological, evolutionary & environmental sciences

For a reference copy of the document with all sections, see nature.com/documents/nr-reporting-summary-flat.pdf

Life sciences study design

All studies must disclose on these points even when the disclosure is negative.

Sample size	No statistical methods were used to predetermine sample size.<br>For mouse experiments, the sample size was chosen according to investigators' prior experience (doi: 10.1093/hmg/ddq468, 10.1172/JCI78448, 10.1210/jc.2018-00646, 10.1172/JCI141587).<br>For in vitro experiments, optimal number of cells and number of technical and biological independent replicates were chosen according to the investigators' prior experience (doi: 10.1172/JCI78448, 10.1159/000508375) and pilot experiments.
Data exclusions	No data were excluded from the analyses.
Replication	Mouse experiments: analysis of GnRH neuron phenotype, puberty onset assessment, gonadal maturation analyses, median eminence loop quantification and Evans Blue permeability were performed across at least three different mice per group. Detailed n numbers are outlined in the text. Expression studies were performed in two independent experiments.<br><br>In vitro assays with COS-7 cells (western blot and immunostaining) were repeated in three independent experiments.<br>In vitro assays with HUVEC and mBEC cells (TEER analysis) had three technical replicates and were repeated in three (HUVECs) and two independent experiments (mBECs and shScr/shPLXNA2 HUVECs) with SEMA6A/mock conditioned media harvested from different COS-7 cell transfection experiments.<br><br>All the attempts at replication were successful.
Randomization	No specific randomization protocols were used for mouse experiments; the experimental groups were determined by genotype.<br>Replicates for HUVEC and mBEC cells belonging to the same treatment group were evenly distributed among different plates used to allocate the transwell supports to avoid any possible plate related bias.
Blinding	Mouse experiments: Investigators were blind to group allocation during data allocation and analysis. Samples were coded at the beginning of the experiment and were only decoded after analysis was completed. During puberty onset assessment analyses, investigators were not blind.<br><br>In vitro experiments: Investigators were not blind to group conditions because the experiments were not evaluated using subjective metrics and therefore not at risk for bias.

Reporting for specific materials, systems and methods

We require information from authors about some types of materials, experimental systems and methods used in many studies. Here, indicate whether each material, system or method listed is relevant to your study. If you are not sure if a list item applies to your research, read the appropriate section before selecting a response.

Materials & experimental systems

n/a	Involved in the study	
[ ]	[X]	Antibodies
[ ]	[X]	Eukaryotic cell lines
[X]	[ ]	Palaeontology and archaeology
[ ]	[X]	Animals and other organisms
[X]	[ ]	Clinical data
[X]	[ ]	Dual use research of concern
[X]	[ ]	Plants

Methods

n/a	Involved in the study	
[X]	[ ]	ChIP-seq
[X]	[ ]	Flow cytometry
[X]	[ ]	MRI-based neuroimaging

Antibodies

Antibodies used	For immunofluorescence staining mouse:<br>mouse anti-nTUBB3 (1:500, clone Tuj1; Covance, MMS-435P); rabbit anti-PRPH (1:100; Merck Millipore, AB1530, lot 2757178); rabbit anti-GnRH (1:400; Immunostar, 20075, lot 1037001); rabbit anti-BLBP (1:200; Millipore, ABN14); goat anti-SEMA6A (1:200; R&D Systems, AF1615, lot JEG012101A and JEG0122071); rabbit anti-SYP (1:200; Synaptic System, 101002); rabbit anti-GFAP (1:500; Dako, Z0334); rabbit anti-OLIG2 (1:300; Millipore, cat. AB9610); mouse anti-VIM (1:200, clone E-5; Santa Cruz Biotechnology, sc-373717, lot

G1916); rabbit anti-GSTP1 (1:200; MBL, 312); rat anti-PDGFRα (1:100, clone APA5; Invitrogen, 14-1401-81, lot 2417022); mouse anti-PLXNA2 (1:100, clone A-2; Santa Cruz Biotechnology, sc-393939, lot F0719); goat anti-PLXNA2 (1:100; R&D Systems, AF5486); rabbit anti-PLXNA4 (1:100; Novus Biologicals, NBP1-85128, lot CFNB011607A); goat anti-PLXND1 (1:200; R&D Systems, AF4160); rat anti-PLVAP antibody (1:100, clone Meca32; BD Biosciences, 550563); rabbit anti-PECAM1 (1:200; Abcam, ab28364); rat anti-PECAM1 (1:200; BioLegend, 102502, lot B321998); biotinylated isolectin B4 (1:100; Vector laboratories, B-1205, lot S0929).

For immunofluorescence staining cells:
mouse anti c-myc (1:500, clone 9E10; ThermoFisher, 13-2500), goat anti-CDH5 (1:200; Santa Cruz Biotechnology, sc-6458, lot G2015); rat anti-PECAM1 (1:200; BioLegend, 102502, lot B321998); rat anti-ICAM2 (1:200, clone 3C4; BD Biosciences, 553326); goat anti-PLXNA2 (1:200; R&D Systems, AF5486). Phalloidin-tetramethyl isothiocyanate (1:400, Sigma Aldrich, P1951-1MG).

Secondary antibodies for immunofluorescence labelling:
Alexa488- or Cy3-conjugated donkey anti-goat Fab fragments (1:200; Jackson Immunoresearch, 705-546-147 and 705-166-147)
Alexa488-, Alexa647- or Cy3-conjugated donkey anti-rabbit Fab fragments (1:200; Jackson Immunoresearch,711-546-152, 711-606-152 and 711-166-152)
Alexa488- or Cy3-conjugated donkey anti-mouse Fab fragments (1:200; Jackson Immunoresearch,715-546-150 and 715-166-150)
Cy3- or Alexa647-conjugated donkey anti-rat Fab fragments (1:200; Jackson Immunoresearch, 712-166-150 and 712-606-150)
Cy3-conjugated streptavidin (1:200; Jackson Immunoresearch, 016-160-084)

For immunoperoxidase labelling:
rabbit anti-GnRH (1:1000; Immunostar, 20075, lot 1037001); rabbit anti-CYP17A1 (1:200; Proteintech, 14447-1-AP, lot 00042070); goat anti-SEMA6A (1:200; R&D Systems, AF1615, lot JEG012101A and JEG0122071).
Secondary antibodies for immunoperoxidase labelling:
anti-rabbit biotinylated antibody (1:400; Vector Laboratories, BA-1000, lot ZB1007); anti-goat biotinylated antibody (1:400; Vector Laboratories, BA-9500). Signal was amplified with the ABC kit (Vector Laboratories, PK-6100).

For immunoblotting:
mouse anti c-myc (1:500, clone 9E10; ThermoFisher, 13-2500), rabbit anti-GAPDH (1:1000; Cell Signaling, 5174S).
Secondary antibodies for immunoblotting:
Goat anti-mouse conjugated HRP (1:4000; Agilent, P044701-2) and goat anti-rabbit conjugated HRP (1:2000; Sigma-aldrich, A4914).

Validation

All antibodies were commercially available and therefore validated by previous investigators. Certificates of analysis for the approved applications and relevant references are provided on manufacturer's website.

- Rabbit anti-PRPH (https://www.merckmillipore.com/IT/it/product/Anti-Peripherin-Antibody,MM_NF-AB1530?ReferrerURL=https%3A%2F%2Fwww.google.com%2F) this anti-Peripherin antibody is validated for use in immunohistochemistry for the detection of Peripherin; strong staining on rat and human. Cross-reacts with mouse, pig, and bovine. Electrophoretically pure trp-E-peripherin fusion protein, containing all but the 4 N terminal amino acids of rat peripherin. Fusion protein purified from bacterial inclusion bodies by DEAE-cellulose chromatography in 6 M urea followed by preparative SDS-PAGE. Routinely evaluated by Western Blot on PC12 lysates.
- Rabbit anti-BLBP antibody (https://www.merckmillipore.com/IT/it/product/Anti-Brain-lipid-binding-protein-Antibody,MM_NF-ABN14) detects Brain lipid binding protein and is validated for use in immunohistochemestry. The epitope recognized is the cytoplasmic domain of the GST-tagged recombinant protein corresponding to human Brain lipid-binding protein. Evaluated by Western Blot in P1 mouse brain tissue lysate.
- Goat anti-SEMA6A (https://resources.rndsystems.com/pdfs/datasheets/af1615.pdf?v=20220618&_ga=2.176133223.413678062.1655563412-1531890473.1655563412) is a polyclonal antigen affinity-purified antibody raised against S. frugiperda insect ovarian cell line Sf 21-derived recombinant mouse Semaphorin 6A (Gly19-Thr649). It detects mouse Semaphorin 6A in direct ELISAs, Western blots and immunohistochemistry. In Western blots, approximately 25% cross�reactivity with recombinant human (rh) Semaphorin 6A is observed and less than 1% cross-reactivity with rhSemaphorin 3A, rhSemaphorin 3B, recombinant mouse (rm) Semaphorin 3C, rmSemaphorin 3F, and rmSemaphorin 7A is observed. It was validated on Sema6a knock out mouse tissues (doi: 10.1182/blood-2012-02-410076).
- Rabbit anti-Gnrh (https://www.immunostar.com/wp-content/uploads/20075-1911001.pdf) is a polyclonal antibody raised against synthetic LHRH coupled to keyhole limpet hemocyanin (KHL) with carbodiimide (CDI) linker. This antibody recognized GnRH of several species included mouse and it is quality control tested for immunohistochemistry. It has been widely employed in the field by us and other researchers as proved by product citations (https://www.citeab.com/antibodies/677104-20075-lhrh-luteinizing-hormone-releasing-hormone?utm_campaign=Widget+All+Citations&utm_medium=Widget&utm_source=ImmunoStar&utm_term=ImmunoStar)
- Goat anti-PLXNA2 (https://resources.rndsystems.com/pdfs/datasheets/af5486.pdf?v=20220618&_ga=2.70729429.413678062.1655563412-1531890473.1655563412) is a polyclonal antigen affinity-purified antibody raised against Chinese hamster ovary cell line CHO-derived recombinant mouse Plexin A2 (Met35-Pro1237). It is recommended for detection of mouse PLXNA2 by western blot, flow cytometry and immunohistochemistry.
- Rabbit anti-PLXNA4 (https://www.novusbio.com/PDFs/NBP1-85128.pdf) is a polyclonal antibody raised against a portion of recombinant PLXNA4 and it was purified by immunogen affinity. It recognizes human, mouse and rat PLXNA4 and it has been validated for immunohistochemistry.
- Goat anti-PLXND1 (https://resources.rndsystems.com/pdfs/datasheets/af4160.pdf?v=20220618&_ga=2.105324486.413678062.1655563412-1531890473.1655563412) is a polyclonal antigen affinity-purified antibody raised against mouse myeloma cell line NS0-derived recombinant human Plexin D1 (Leu47-Ala1271), which shares 89% identity with mouse Plexin D1. It recognizes human PLXND1 but it has been successfully tested on mouse brain tissues for the detection of GnRH neurons (doi: 10.1172/JCI78448).
- Goat anti-VE-Cadherin (https://datasheets.scbt.com/sc-6458.pdf) this is an affinity purified goat polyclonal antibody raised against a peptide mapping at the C-terminus of VE-cadherin of human origin. It is recommended for detection of VE-cadherin of mouse, rat and human origin by immunofluorescence
- Rat anti-CD31 (https://www.biolegend.com/de-at/products/purified-anti-mouse-cd31-antibody-380?pdf=true&displayInline=true&leftRightMargin=15&topBottomMargin=15&filename=Purified%20anti-mouse%20CD31%20Antibody.pdf) is an affinity isolated antibody purified by affinity chromatography, it recognizes its respective epitope in IgD2 of

CD31. Each lot of this antibody is quality control tested by immunofluorescent staining with flow cytometric analysis.
- Rat anti-PLVAP (https://www.bdbiosciences.com/en-nz/products/reagents/flow-cytometry-reagents/research-reagents/single-color-antibodies-ruo/purified-rat-anti-mouse-panendothelial-cell-antigen.550563) is an affinity chromatography-purified monoclonal antibody (clone MECA-32) obtained from tissue culture supernatant or ascites and it is raised against mouse lymph node stromal cells. The MECA-32 antibody specific for mouse panendothelial cell antigen is recommended to test for immunohistochemical staining.
- Rabbit anti-SYP (https://sysy.com/product/101002) is a polyclonal antiserum raised against a synthetic peptide corresponding to residues near the carboxy terminus of human Synaptophysin. It reacts with human, rat and mouse synaptophysin and it has been widely employed to detect mouse SYP by IHC on frozen tissues.
- Rabbit anti-GFAP (https://www.agilent.com/en/product/immunohistochemistry/antibodies-controls/primary-antibodies/glial-fibrillary-acidic-protein-(concentrate)-76683#specifications) is a purified polyclonal antibody raised against GFAP isolated from cow spinal cord. The antibody shows distinct precipitate with cow brain extract and no reaction with human and cow serum by indirect ELISA. It is recommended for detection of human GFAP by immunohistochemistry but is has been successfully tested on mouse brain tissues (doi: 10.1016/j.neuron.2022.07.010).
- Mouse anti-VIM (https://www.scbt.com/it/p/vimentin-antibody-e-5) is a monoclonal antibody specific for an epitope mapping between amino acids 411-447 near the C-terminus of human VIM. It is suitable for detection of mouse, rat and human detection of VIM by western blot and immunofluorescence.
- Rabbit anti-OLIG2 (https://www.merckmillipore.com/IT/it/product/Anti-Olig-2-Antibody,MM_NF-AB9610) is a purified polyclonal antibody raised against mouse recombinant OLIG2 and is recommended for detection of human, mouse and rat OLIG2 by immunohistochemistry.
- Rabbit anti-GSTP1 (https://www.mblbio.com/bio/g/dtl/A/index.html?pcd=312) is an affinity purified polyclonal antibody raised against purified human GSTP1. It is recommended for the detection of human, mouse, rat and hamster GSTP1 by immunofluorescence.
- Rat anti-PDGFRα (https://www.thermofisher.com/antibody/product/CD140a-PDGFRA-Antibody-clone-APA5-Monoclonal/14-1401-82) is a monoclonal antibody (clone APA5) purified by affinity chromatography. The APA5 antibody has been reported for use in flow cytometric analysis, immunoblotting (WB), and immunohistochemical staining of frozen tissue sections and recognizes the alpha chain of the platelet derived growth factor receptor (CD140a).
- Mouse anti-PLXNA2 (https://www.scbt.com/it/p/plexin-a2-antibody-a-2) is a monoclonal antibody (clone A-2) specific for an epitope mapping between amino acids 1159-1196 within an internal region of plexin-A2 of human origin. It is recommended for the detection of human, mouse and rat PLXNA2 by immunofluorescence.
- Rat anti-ICAM2 (https://www.bdbiosciences.com/en-nz/products/reagents/flow-cytometry-reagents/research-reagents/single-color-antibodies-ruo/purified-rat-anti-mouse-cd102.553326) is a monoclonal antibody raised against a recombinant protein produced in transfected cell lines. It reacts with mouse ICAM2 (QC tested) and it is routinely testes by flow cytometric analysis.
- Rabbit anti-PECAM1 (https://www.abcam.com/products/primary-antibodies/cd31-antibody-ab28364.html) is a polyclonal antibody raised against the synthetic peptide within mouse CD31 aa 700 to the C-terminus (C terminal). It recognizes human CD31 by immunohistochemistry and is predicted to work with mouse. It has been successfully used to detect mouse PECAM1 in frozen brain tissues (doi: 10.1038/mtm.2015.37 and doi: 10.1186/s13041-021-00862-y).
- Rabbit anti-CYP17A1 (https://www.ptglab.com/products/CYP17A1-Antibody-14447-1-AP.htm#product-information) is a polyclonal antibody raised against a CYP17A1 fusion protein Ag5899. It is suitable for the detection of human, mouse and rat cytochrome P450, family 17, subfamily A, polypeptide 1.
- Mouse anti c-myc (https://www.thermofisher.com/order/genome-database/dataSheetPdf?producttype=antibody&productsubtype=antibody_primary&productId=13-2500&version=233) is a monoclonal antibody raised against a 32 amino acid synthetic peptide (aa 408-439) derived from the C-terminus of the human c-myc protein ant it is recommended for western blot and immunocytochemistry.
- Rabbit anti-GAPDH (https://www.cellsignal.com/datasheet.jsp?productId=5174&images=1&size=A4) is a monoclonal antibody produced by immunizing animals with a synthetic peptide corresponding to residues near the carboxy terminus of human GAPDH. It is recommended for detection of mouse, human, rat and monkey GAPDH by western blot and has more than 4000 citations.

Eukaryotic cell lines

Policy information about cell lines and Sex and Gender in Research

Cell line source(s)	COS-7 cells (American Type Culture Collection, cat. CRL-1651)<br>HUVECs (Promocell, cat. FB60C12203)<br>Mouse brain endothelial cells (mBECs) were isolated from brains of adult WT C57/Bl6 mice as previously described (doi: 10.1371/journal.pone.0226302).
Authentication	COS-7 and HUVECs have been purchased and therefore authenticated by the suppliers. Specifically, HUVECs were tested for cell morphology, adherence rate, and cell viability and flow cytometric analyses for cell-type specific markers, e.g. CD31 and Dil-Ac-LDL uptake are also carried out for each lot.<br>mBECs were not authenticated; purity of mBECs was assessed by immunostaining for PECAM1 and CDH5.
Mycoplasma contamination	COS-7 and HUVEC cell lines were tested negative for mycoplasma contamination by the suppliers.<br>mBECs are primary cells and have been cultured only for the experimental time-frame. Thus, mBECs were not tested for mycoplasma contamination.
Commonly misidentified lines<br>(See ICLAC register)	No commonly misidentified cell lines were used in this study.

Animals and other research organisms

Policy information about studies involving animals; ARRIVE guidelines recommended for reporting animal research, and Sex and Gender in Research

Laboratory animals	HA-Sema6afl/fl mice which harbor a 3xHA tag in exon 2 of Sema6a and lox sites surrounding exon 3, were a kind gift of Alex Kolodkin (Johns Hopkins University School of Medicine, Baltimore, USA). Germline deletion of Sema6a exon 3 was performed by crossing these mice with EIIa-Cre mice (JAX stock #003724, doi: 10.1073/pnas.93.12.5860) to create Sema6a-null mice (here referred to as Sema6a-/-) that were maintained on a C57Bl/6J background (JAX strain code 632; Charles River). For expression studies on wild-type mouse tissues, C57Bl/6J pregnant mice were acquired from Charles-River.<br>Animal experiments were carried out on animals aged embryonic day (E) 12.5, E14.5 and E18.5 or postnatal day (P) 24, P30 and P60. The morning when a plug was observed is considered as E0.5 and the day of birth is defined as P0.<br>Mice were housed in groups, with access to food (chow diet CRM(E) SQC811437, Special Diet Services) and water ad libitum on a 12 hour light/dark cycle, humidity of 45-65% and a temperature between 20-24 degrees.<br>Timed pregnant females were sacrificed by cervical dislocation to collect embryos at E12.5, E14.5 and E18.5. Embryo heads were fixed for 3-6 h in 4% PFA at 4°C. Adult mice were euthanized by overdosing Euthanimal (200 mg/mL, Alfasan) intra-peritoneal (i.p.), and transcardially perfused with ice-cold 4% PFA.
Wild animals	No wild animals were used in this study
Reporting on sex	Mice: embryos were not genotyped for sex. Both female and male adult wildtypes or sema6a mutants were studied for assessing fertility and GnRH neuron phenotypes. Only female mice were used for permeability analyses. Sex was not assessed for expression studies on wild-type adult mice.
Field-collected samples	No field-collected samples were used in this study.
Ethics oversight	All in vivo experiments were conducted in compliance with the Animal Ethics Committee of Utrecht University (Dierexperimenten Ethische Commissie) (CCD licence: AVD115002016532) and the Animal Welfare Body of the University of Milan, and in agreement with Dutch laws (Wet op de Dierproeven, 1996; revised 2014), the Italian Minister of Health and European regulations (Guideline 86/609/EEC; Directive 2010/63/EU).

Note that full information on the approval of the study protocol must also be provided in the manuscript.
